# Comparison of the Marginal Integrity of Sectional Non-Invasive Laminate Veneers Versus Sectional Minimally Invasive Laminate Veneers Under Artificial Aging

**DOI:** 10.3390/dj13080358

**Published:** 2025-08-07

**Authors:** Polykarpos Papanagiotou, Phophi Kamposiora, George Papavasiliou, Spiros Zinelis

**Affiliations:** 1 Department of Prosthodontics, Dental School, National and Kapodistrian University of Athens, 11527 Athens, Greece; polpapan@gmail.com (P.P.); phophik@odontiki-anaplasi.gr (P.K.); geopap@dent.uoa.gr (G.P.); 2 Department of Biomaterials, School of Dentistry, National and Kapodistrian University of Athens, 11527 Athens, Greece

**Keywords:** veneers, dental porcelain, lithium disilicate

## Abstract

**Background/Objectives**: To compare the marginal integrity of sectional non-invasive laminate veneers versus sectional minimally invasive laminate veneers. **Methods**: A total of eighty (80) intact maxillary and mandibular frontal teeth (central incisors) were randomly divided into four groups (*n* = 20). Two groups received non-invasive veneers made of feldspathic porcelain (Feldspathic Non-Invasive—FNI) and lithium disilicate (Lithium Disilicate Non-Invasive—LNI) accordingly. Groups FP and LP received minimally invasive veneers manufactured by feldspathic porcelain and lithium disilicate, respectively. Following cementation, three grooves with mesio-distal orientation on the labial surface of teeth of each sample, at the incisal, middle, and cervical third, were made. Before and after artificial aging, the mesio-distal distance between the end of the groove on the tooth and the edge of each veneer was measured in micrometers (μm) employing an SEM immediately after cementation (T_0_), after simulated artificial aging equivalent to four months of everyday brushing (T_1_), and after twelve months of everyday brushing (2 times per day) (T_2_) to identify the wear of veneers. In the same manner, the horizontal dimension of the cement layer extending from its edge till the margin of the veneer was measured for all the groups at T_0_, T_1_, and T_2_, respectively. The statistical analysis was performed employing non-parametric Kruskal–Wallis ANOVA and Dunn’s test. **Results**: No significant differences from T_0_ to T_1_ and from T_1_ to T_2_, as well as from T_0_ to T_2_, were identified for all the groups tested. No significant differences were allocated among all groups for the dimensional changes in the cement. **Conclusions**: All the groups responded similarly to aging factors, regardless of the non-invasive or minimally invasive approach, or the material used to fabricate the veneers.

## 1. Introduction

Porcelain veneers were introduced to the profession by John Calamia of New York University, USA, in the early 1980s [1]. Since then, a lot of effort has been made to improve the optical characteristics of the ceramic materials, the bond strength with tooth structure, and the esthetic outcome of veneer restorations [1,2,3,4,5]. However, clinical success and endurance of porcelain veneers are challenging due to the variety of factors involved. Marginal and internal fit [6,7,8,9,10], combined with adequate ceramic thickness [11,12,13], tooth preparation design [14,15,16,17,18], and most importantly, cementation [19,20,21,22], are factors that affect the success of the treatment. Gentle handling during the clinical try-in procedures [23], precise seating of the veneer, and control of the cement excess [24] are crucial to avoid marginal fractures, excessive marginal gaps, microleakage, and secondary caries due to malposition [19,20,21,22,25].

At the same time, the clinician depends on the technician’s expertise to fabricate a prosthesis that fulfills not only the esthetic expectations of the patient but also the mechanical demands during function. Thus, the manufacturing technique [7,26,27,28,29,30,31,32,33], the type of ceramic material [26,34,35] are of paramount importance for the clinical efficacy of these restorations.

In the last few years, minimally invasive dentistry has been introduced as a promising solution for closing diastemas in the frontal area [8,36,37,38,39]. The concept of preserving tooth structure by non-invasive veneers has many advantages. During the treatment, there is no need for anesthesia, the periodontal tissue is not disturbed by impression cords, and provisional restorations are not necessary [40,41,42]. The patient experiences minimal discomfort during the clinical steps and a pain-free transition from the unaesthetic tooth diastema to a beautiful smile. Up to now, there are studies that evaluate sectional non-invasive veneers under aging factors both in vitro and in vivo, but there are no data to compare sectional invasive and non-invasive veneers [40,41,43,44,45,46].

A relevant clinical question is whether non-invasive or minimally invasive veneers can be a reliable solution for closing diastemas in the frontal area. The aim of this comparative study is to test the marginal integrity of sectional non-invasive and minimal invasive laminate veneers ex vivo under simulated aging factors. The null hypothesis is that marginal integrity is not affected either by the clinical approach (non-invasive or minimally invasive) or by the materials used for the fabrication of the veneers.

## 2. Materials and Methods

All materials (Table 1) used in this comparative study were acquired by one manufacturer and were handled according to the manufacturer’s instructions. A total of eighty (80) intact maxillary and mandibular frontal teeth were included in this study. The selected teeth were extracted due to aggressive periodontitis, orthodontic treatment, or surgical reasons. The research protocol has been approved by the ethics committee of the Dental School of the National and Kapodistrian University of Athens. (Ethics approval number: 295-09/03/2016).

The teeth were stored in physiological saline solution [22]. Subsequently they were cleaned with hand scaling to remove calculus and periodontal fibers and were examined with magnification loupes at 2.3× nominal magnification to assess tooth structure. The teeth were randomly divided into four (4) groups (*n* = 20/group) with the following characteristics:

Group FNI received non-invasive laminate veneers, made of feldspathic porcelain

Group LNI received non-invasive laminate veneers, made of lithium disilicate.

Group FP received minimally invasive laminate veneers, made of feldspathic porcelain.

Group LP received minimally invasive laminate veneers, made of lithium disilicate.

Teeth preparation: All the teeth samples from Group FP and Group LP underwent partial veneer preparation. The veneer preparation was completed with a water-cooled high-speed handpiece (Kavo, Gentlesilence LUX 6500B, Biberah, Germany). A diamond bur system specific to ceramic veneers (Komet, REF 4151, Lemgo, Germany) was used to create the necessary space for the veneer. The outline form of each partial veneer extended from the midline of the labial surface to the mesial or the distal surface of the tooth, and 2 mm above the CEJ up to the incisal edge of the tooth. The preparation was initiated by depth cutter burs (Komet, REF4151, CVS-1) to define the appropriate depth of 0.6 mm, followed by a chamfer bur (Komet, REF4151, CVS-4) to refine the preparation. The incisal edge was not prepared. Group FNI and Group LNI received sectional non-invasive veneers, and thus, no preparation was performed.

Fabrication of the porcelain veneers: A duplicate model was fabricated for each specimen. A single dental technician fabricated all the necessary veneers using either feldspathic porcelain (IPS e.max Ceram, Ivoclar Vivadent AG, Schaan, Liechtenstein) or IPS e.max Press ingots (Ivoclar Vivadent AG). Group FNI and group FP received feldspathic porcelain veneers. The refractory dyes technique (Nori-Vest; Kuraray Noritake Dental Inc., Hattersheim am Main, Germany) was used to manufacture feldspathic veneers. This technique permitted the use of layers with multiple levels of opacity, claiming enhanced esthetics [34,47,48,49,50]. Pressed lithium disilicate (IPS e.max Press ingots manually generated with staining) was used to fabricate the restorations for group LNI and LP. Following the recommendations of the manufacturer, pressed lithium disilicate is recommended for laminate veneers with a minimum preparation of 0.6 mm.

**Figure 1:**
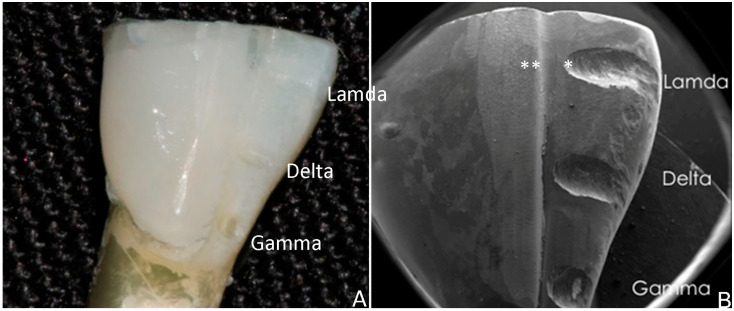
(**A**) Stereomicroscope image of prepared teeth (nominal magnification 3×) and (**B**) Secondary Electron Image from the surface of prepared teeth (nominal magnification 6×). The initial point (edge of the notch) and the final point (edge of the veneer) are indicated by one and two asterisks, respectively.

Cementation: Prior to cementation, all the teeth samples were cleaned with a micromotor brush using a fluoride-free cleaning paste (Proxyt, RDA 36, Ivoclar Vivadent AG). The paste was removed with water spray and oil-free air. A self-etching glass-ceramic primer (Monobond, Ivoclar Vivadent AG) was applied on the intaglio surface of the veneer with a micro brush for twenty seconds and allowed to react for forty seconds. Then, the primer was removed with water spray and oil-free air for ten seconds. A phosphoric acid gel (Total Etch, Ivoclar Vivadent AG) was applied on the designated labial area of the tooth and reacted for thirty seconds. The gel was removed with water spray and oil-free air. In the same area, a light-cured adhesive (Adhese Universal Vivapen, Ivoclar Vivadent AG) was applied for twenty seconds. Excess adhesive was removed with gentle air spray. The adhesive was then light-cured for ten seconds. A light-curing luting composite (Variolink Esthetic LC, Ivoclar Vivadent AG) of translucent color was applied on the intaglio surface of the veneer, and the restoration was seated on the designated area of the tooth by means of an adhesive tip applicator (Optra Stick, Ivoclar Vivadent AG). While seated, the excess cement was light-cured for ten seconds following the margin line and was removed with a scaler without damaging the veneer. To prevent oxygen inhibition, a glycerine gel (Liquid Strip, Ivoclar Vivadent AG) was applied on the margin and light-cured for ten seconds. The gel was rinsed with water spray. Finally, the margin of the veneer was polished with a diamond polishing system (OptraFine, Ivoclar Vivadent AG). Following cementation, a thin diamond bur was used to make three (3) horizontal grooves on the labial surface of each sample, at the incisal, middle, and cervical third. These grooves were used as reference points for future observations under the SEM. (Figure 1).

Artificial Aging Regime: All the specimens were exposed to aging procedures, including thermocycling and brushing, in order to age the veneers in a similar way to the clinical situation. The specimens underwent thermocycling (ISO 11405) at 5000 cycles between two water baths with distilled water of 5 °C and 55 °C with a dwell time of 60 s in a custom-made thermocycling machine. A robotic arm was used to immerse the specimens in two baths for the preset dwell time. After thermocycling the specimens were rinsed with distilled water and allowed to dry in ambient conditions. The aging through brushing was simulated following Fones’ Rotary technique using an electric powered toothbrush (Philips Sonicare DiamondClean 9000, Philips, Amsterdam, The Netherlands) and a toothpaste (Colgate Total, Colgate-Palmolive, Swidnica, Poland) [48,49,50,51,52,53,54]. A custom-made device was used to stabilize the tooth position on a horizontal plane, and the brushing procedure was implemented by a single operator by mixing toothpaste with tap water. The operator was brushing in a continuous cycling motion mimicking the brushing technique. For each specimen a period of thirty (30) seconds of brushing per day was estimated. All the specimens underwent brushing aging equivalent to a duration of four months initially and twelve months in total. All specimens were subjected to 10,000 strokes simulating approximately one year of oral aging [55].

Measurements: All the specimens were subjected to Scanning Electron Microscope (Quanta Inspect D8334, FEI, Hillsboro, OR, USA) observation, at three stages:

1. Immediately after cementation (T0).

2. Following simulated artificial aging equivalent to four months (T1).

3. Following simulated artificial aging equivalent to twelve months (T2).

**Figure 2:**
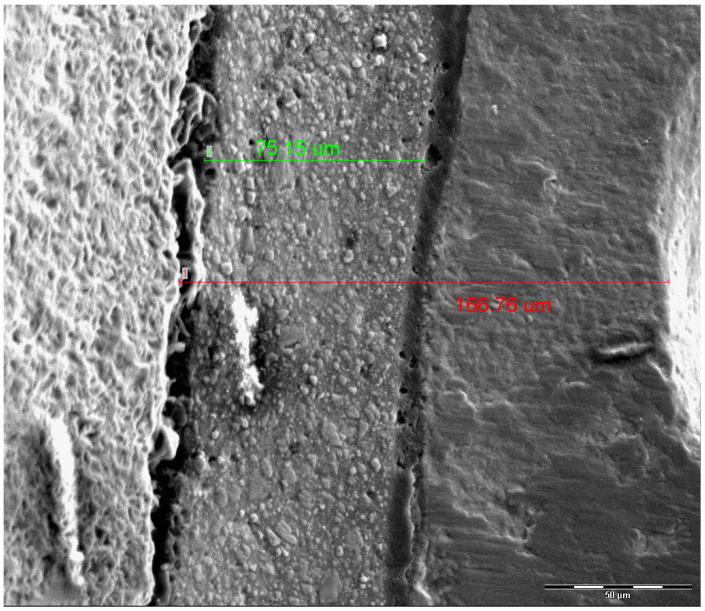
Measurement of mesio-distal distance between the groove and the edge of the veneer (red line) and the extent of the cement layer (green line). Nominal magnification 500×, scale bar: 50 μm.

At T0, the horizontal grooves (incisal, middle, cervical) of the labial surface were examined in relationship with the margin and the cement layer. One of the grooves was randomly selected employing a free online randomization software (https://www.randomizer.org, accessed on 16 March 2023). Henceforth, for each specimen, that area of the labial surface was examined, and the groove included in the selected area will define the area of interest until the end of the observation period [56]. The specimens were placed on aluminum stubs, sputter-coated with gold (Emitech SC7620 Sputter Coater; Emitech, Paris, France) and imaged by Secondary Electron Detector employing high vacuum conditions, operating at 15 kV, 98 μA beam current, and observed at 500× nominal magnification. Secondary Electron Images (SEI) were taken from the selected area. The horizontal distance between the end of the groove and the edge of each veneer (in the mesio-distal orientation) was measured in micrometers (μm) employing the dedicated image analysis software (XT Docu ver3.2; Soft Imaging System GmbH, FEI) as shown in Figure 2. The measurements took place at T0, T1, and T2, respectively. The teeth were placed in the SEM stage with the same orientation, and the stored image at T0 was used to identify the reference point at the edge of the groove (Figure 2). In the same manner, the mesio-distal distance of the cement layer extending from its edge till the margin of the veneer was measured for all the groups at T0, T1, and T2, respectively.

### Statistical Analysis

All the results were initially tested for the presence of outliers employing the Grubbs test. Then the groups were first tested for normality using the Kolmogorov–Smirnov criterion. Quantitative variables were presented with mean, median, and 25% and 75% quartiles as normality check failed. Statistically significant differences were identified by non-parametric Kruskal–Wallis ANOVA and Dunn’s multiple comparison test. In all the cases the statistical level of significance was set at α = 0.05. The statistical analysis was carried out by OriginPro 2021 v. 9.8 (OriginLab Corporation, Northampton, MA, USA).

## 3. Results

**Figure 3:**
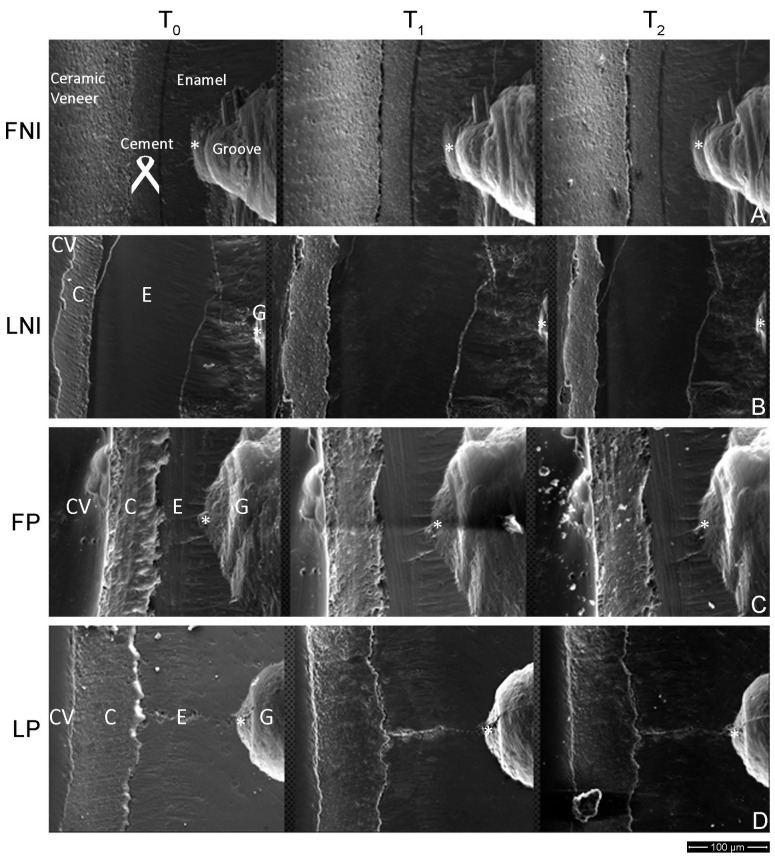
Successive SE images from the same region for all the groups tested at T_0_, T_1_, and T_2_. (**A**) FNI, (**B**) LNI, (**C**) FP, and (**D**) LP. All the reference points are indicated by an asterisk. Ceramic venners (CV), cement (C), enamel (E), and groove (G) are indicated on the left images. Nominal magnification 500×, scale bar: 100 μm.

**Figure 4:**
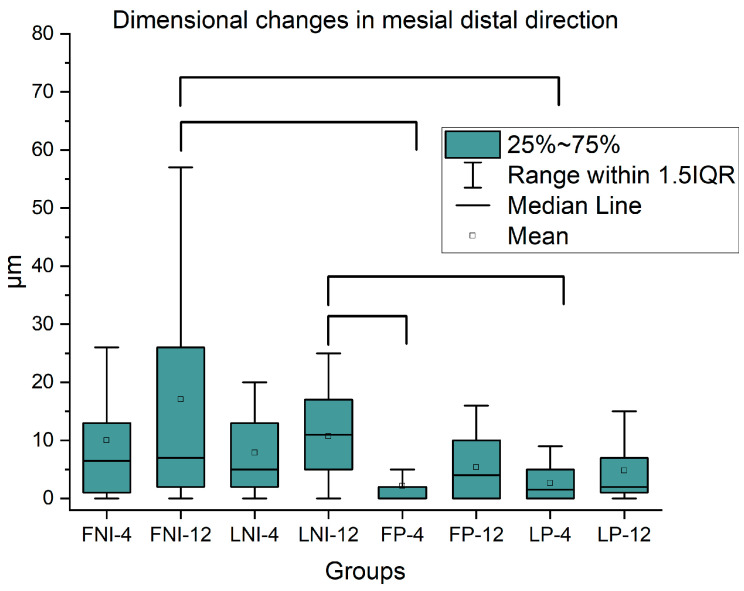
Box plots including mean, median, and 25% and 75% percentiles of dimensional changes in the mesial–distal direction for all the groups tested (*n* = 20). Brackets connect mean values with statistically significant differences (*p* < 0.05).

Figure 3 presents successive images from the same region of interest for all the groups tested at T0, T1, and T2. Different regions are identified on the left image of each row, indicating the ceramic veneer, the cement, the enamel, and the groove areas, while Figure 4 shows the dimensional changes in the mesial–distal direction with mean, median, 25% and 75% percentiles. Brackets connect mean values with statistically significant differences (*p* < 0.05).

**Figure 5:**
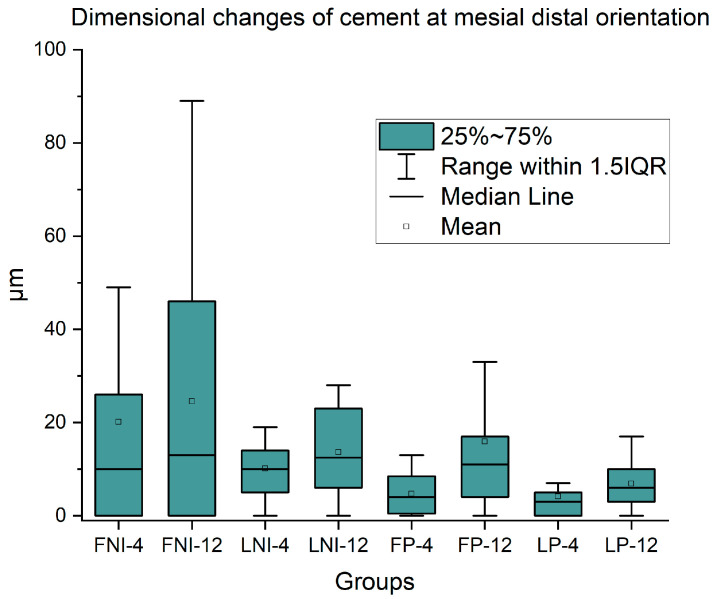
The dimensional changes in cement in the mesial–distal orientation with mean, median, and 25% and 75% percentiles are presented (*n* = 20). No statistically significant differences were found for all the groups tested (*p* > 0.05).

During the follow-up period, mesio-distal distance from the notch to the margin of the veneer was increased for all under-study groups, indicating wear of the veneer. However, no significant differences were identified from T0 to T1, from T1 to T2, as well as from T0 to T2 for all the groups tested. In Figure 5 the dimensional changes in the cement in the mesial–distal orientation with mean, median, 25%, and 75% percentiles are presented. In all the measurements, there were non-significant differences among all the groups under study (*p* > 0.05).

## 4. Discussion

The results of the study confirm both parts of the null hypothesis. Regarding the first part, all the groups had similar performance to aging factors with no significant differences between the non-invasive and minimally invasive approaches. For the second part, both materials used to fabricate the veneers responded evenly to the aging factors with no significant differences between them, and thus both null hypotheses should be accepted.

Water thermocycling was used to simulate the wet oral environment and the effect of temperature fluctuations at the interfaces of different materials (enamel, cement, porcelain) with differences in coefficients of thermal expansion. In clinical circumstances, when sectional non-invasive veneers are used to close diastemas on the frontal area, it is common for clinicians to correct interferences and thus avoid any force load applied on the veneer [45,46,47]. At the same time, patients are instructed to avoid extensive biting force over veneered teeth but not to neglect brushing them. Brushing of teeth is the most common way of oral care, and this is why it was selected as an aging factor in the study. Patients are educated by dentists to brush their teeth at least two times per day as a standard of care, and the toothbrush is the most recommended product to do so [48,49]. A power toothbrush was chosen over a manual one because it is commonly used by the public [49,54]. Nowadays, power toothbrushes are strongly commercially promoted, and thus more patients tend to embed them in their daily oral care routine. Natural teeth rather than models were chosen in the study to utilize their natural, physical, and mechanical properties, bonding capacity, and geometrical characteristics, eliminating differences from real clinical situations [11,40]. Instead of multiple clinicians participating, only one operator was handling the try-in procedures and the cementation of the veneers in an effort to minimize the effect of human factor on the outcome of this study [23]. SEM was used to document the marginal integrity of the samples under an artificial aging regime. This approach has the advantage of obtaining detailed information on the areas of interest, keeping intact the tooth surface, and analyzing the samples in a successive manner, a common methodology for this type of experimental characterization [6,56]. Three different areas were chosen in order to homogenize the possible differences in wear among cervical, middle, and incisal regions.

The specific aging periods of simulated 4 and 12 months were selected to match with first (common 4 to 6 months) and annual recall. During the evaluation period, none of the groups tested presented fracture, debonding, crack lines, or any sign of macroscopically detected surface deterioration (wear, loss of gloss, etc.) due to artificial aging. This is in accordance with previous studies where no significant wear was detected on ceramic and composite materials under the abrasive action of toothbrushing [49,50,51,52,53]. It is interesting to point out that their focus area was at the center surface of the material and not on its margins, as the latter are thinner and sharper and thus more vulnerable to wear phenomena. On the macroscopical level, the marginal deterioration of both groups was not adequate enough to create a difference that a clinician could detect at recall examination using a probe. Contrarily, on microscopic evaluation under an SEM, all the groups showed a marginal deterioration of a few microns but without statistically significant differences either between restorative materials or restorative approach, verifying both null hypotheses. In Figure 4 a few groups showed statistically significant differences after one-way ANOVA, but these comparisons would not be included in a typical 2-way ANOVA as they comprise different materials and time periods (i.e., FNI-12 with NP-4), and thus, no comparison is feasible. This complication arises from the absence of non-parametric two-way ANOVA (non-parametric tests are necessary for populations without normal distributions), and thus one-way ANOVA was the only option for statistical comparison. On a parallel observation, the cement used for the cementation of the veneers behaved in a similar manner during the whole observation period, with no significant differences between the groups. The absence of statistically significant differences is expected as the same material was used for all the groups tested, and thus, the same response to artificial aging was anticipated. Noteworthy to mention that despite the gradual loss of its layer, it delivered an acceptable clinical outcome.

Despite the efforts to simulate oral conditions as much as possible, this study is not free from the inherent limitation of experimental studies where oral parameters (chewing loading, pH fluctuations, irradiation, food consumption, and others) can not be totally simulated. In addition, it should be noted that the absence of significant differences does not imply equivalence, as more data is required to verify this hypothesis from a statistical standpoint of view. The results of this study showed that sectional non–invasive veneers can be a promising option for closing tooth diastemas, providing alternative solutions to esthetic demands. However, more data based on clinical studies, including clinical success, survival, chipping, color stability, etc.) should be collected to enlighten our picture for the clinical efficacy of sectional non-invasive veneers as a promising, safe, and efficient candidate for everyday practice.

## 5. Conclusions

Under the limitations of this study, the following conclusions can be drawn:

The effect of aging factors included in this study (water thermocycling and brushing) is not dependent on either invasive protocol (Sectional non-invasive and minimally invasive) or materials used for the fabrication of veneers.

Sectional non-invasive and minimally invasive veneers responded similarly to aging factors such as brushing and thermocycling for a simulated aging duration of twelve months.

Both materials used to fabricate the veneers, namely feldspathic porcelain and lithium disilicate, performed equally through the testing period regardless of the minimally invasive or non-invasive approach.

The cement used for the cementation was prepared in a similar manner for all the groups throughout the whole testing period.

## Figures and Tables

**Table 1:** Brand names and lot numbers of materials included in this study.

MATERIALS ^1^	LOT
IPS e.max ceram	684725
IPS e.max press ingots HT	626320
Proxyt, RDA 36	701472
Monobond Etch & Prime	626221AN
Total Etch	550588AN
Adhese Universal Vivapen	663720WW
Variolink Esthetic LC cement	666127WW
Liquid Strip	532505AN
Optrafine paste	602289AN

^1^ manufacturer: Ivoclar Vivadent AG.

## Data Availability

Dataset available on request from the authors.
